# The impact of small food workshops management regulations on aflatoxin B_1_ in home-made peanut oil and the liver function of high-consumption area residents: an interrupted time series study in Guangzhou, China

**DOI:** 10.3389/fpubh.2024.1484414

**Published:** 2024-12-20

**Authors:** Jiangbo Lei, Yan Li, Yanyan Wang, Jinchang Zhou, Yuzhe Wu, Yuhua Zhang, Lan Liu, Yijun Ou, Lili Huang, Sixuan Wu, Xuanya Guo, Lieyan Liu, Rongfei Peng, Zhijun Bai, Weiwei Zhang

**Affiliations:** ^1^School of Public Health, Guangdong Pharmaceutical University, Guangzhou, China; ^2^Department of Foodborne Diseases and Food Safety Risk Surveillance, Guangzhou Center for Disease Control and Prevention, Guangzhou, China; ^3^Department of Public Health, Xiaolou Town Health Center, Guangzhou, China; ^4^School of Public Health, Jinan University, Guangzhou, China; ^5^Department of Physical and Chemical Inspection, Guangzhou Center for Disease Control and Prevention, Guangzhou, China

**Keywords:** small food workshop, home-made peanut oil, liver function, aflatoxin B_1_, interrupted time series analysis

## Abstract

**Background:**

Aflatoxin B_1_ (AFB_1_), a potent carcinogen produced by *Aspergillus* species, is a prevalent contaminant in oil crops, with prolonged exposure associated with liver damage. Home-made peanut oil (HMPO) produced by small workshops in Guangzhou is heavily contaminated with AFB_1_. Despite the enactment of the Small Food Workshops Management Regulations (SFWMR), no quantitative assessment has been conducted regarding its impact on food contamination and public health. The study aims to assess the impact of SFWMR on AFB_1_ contamination in HMPO and liver function in the population.

**Method:**

AFB_1_ contamination in HMPO were quantified using high-performance liquid chromatography and liver function data were obtained from the health center located in a high-HMPO-consumption area in Guangzhou. Interrupted time series and mediation analyses were employed to assess the relationship between the implementation of SFWMR, AFB_1_ concentrations in HMPO, and liver function among residents.

**Result:**

The AFB_1_ concentrations in HMPO were 1.29 (0.12, 6.58) μg/kg. The average daily intake of AFB_1_ through HMPO for Guangzhou residents from 2010 to 2022 ranged from 0.25 to 1.68 ng/kg bw/d, and the Margin of Exposure ranged from 238 to 1,600. The implementation of SFWMR was associated with a significant reduction in AFB_1_ concentrations in HMPO, showing an immediate decrease of 2.865 μg/kg (*P* = 0.006) and a sustained annual reduction of 2.593 μg/kg (*P* = 0.034). Among residents in the high-HMPO-consumption area, the implementation of SFWMR was significantly associated with a reduction in the prevalence of liver function abnormality (PR = 0.650, 95% CI: 0.469–0.902). Subgroup analysis revealed that this reduction was significantly associated with the implementation of SFWMR in the female (PR = 0.484, 95% CI: 0.310–0.755) and in individuals aged ≥ 60 years (PR = 0.586, 95% CI: 0.395–0.868). Mediation analysis demonstrated that AFB_1_ concentrations in HMPO fully mediated the relationship between the implementation of SFWMR and the liver function abnormality (PR = 0.981, 95% CI: 0.969–0.993).

**Conclusion:**

In Guangzhou, the public health issue arising from AFB_1_ intake through HMPO warrants attention. The implementation of SFWMR had a positive impact on the improvement of AFB_1_ contamination in HMPO and the liver function. Continued efforts are necessary to strengthen the enforcement of the regulations. The exposure risks to AFB_1_ among high-HMPO-consumption groups also demand greater focus.

## 1 Introduction

Aflatoxins (AFs) are a group of secondary metabolites produced by *Aspergillus* species (primarily *Aspergillus flavus* and *Aspergillus parasiticus*) (1), with aflatoxin B_1_ (AFB_1_) being particularly notable due to its widespread presence and toxicity (2). Approximately 4.5 billion people worldwide are exposed to aflatoxin-contaminated food, particularly in low and middle-income countries in subtropical regions (3, 4). The 2004 outbreak of acute aflatoxicosis in Kenya was among the most significant epidemics of human aflatoxin poisoning recorded in mycotoxin history (5). Tanzania had experienced hundreds of cases of aflatoxicosis in the districts of Kiteto, Chemba, and Kondoa for the three consecutive years since 2016 (6). AFB_1_ is known for its potent carcinogenic properties and is classified as a Group I carcinogen by the International Agency for Research on Cancer (7). The physicochemical stability of AFB_1_ renders it resistant to degradation at conventional cooking temperatures (8), making dietary intake the primary route of human exposure (9). Furthermore, due to its lipophilic nature, foods rich in fat content, such as peanuts and corn, are more susceptible to fungal contamination and subsequent AFB_1_ production (10).

In China, peanuts are the primary oil crop, with ~52% of the total yield allocated for oil extraction, and peanut oil accounts for over 25% of the annual vegetable oil production (11). The consumption of peanut oil in China exhibits distinct regional preferences, primarily in the East China and South China (12). Guangdong Province leads South China with a per capita daily peanut oil consumption of 19.43 grams (13). Local customs and dietary habits have contributed to the widespread presence of small food workshops producing and selling peanut oil across various regions of Guangdong Province (14, 15). Home-made peanut oil (HMPO) is an edible vegetable oil produced operating in rudimentary facilities by private workshops. Unlike industrially processed peanut oil, HMPO retains more of the natural flavor of peanuts and is therefore a preferred choice among consumers. However, the absence of refining, detoxification processes, and product inspection increases the risk of AFB_1_ contamination in HMPO (16).

The effects of AFB_1_ on liver function are primarily characterized by direct hepatocyte damage and impaired energy metabolism (17). AFB_1_ impacts liver function through multiple mechanisms. First, it disrupts mitochondrial bioenergetics and membrane potential (18), promotes mitochondrial cholesterol transport (19, 20), and induces mitochondrial autophagy (21), cumulatively leading to mitochondrial dysfunction. Mitochondrial dysfunction subsequently triggers excessive reactive oxygen species production and compromises antioxidant defenses, intensifying oxidative stress in hepatocytes (22). Additionally, experimental evidence suggests that AFB_1_ contributes to hepatic inflammation via dysregulated intestinal flora (23), release of damage-associated molecular patterns and cytokines, and immune cell activation (24). These mechanisms directly damage hepatocytes and indirectly impair liver function by disrupting overall hepatic metabolic processes.

Long-term consumption of AFB_1_ contaminated HMPO is associated with liver damage (25). In regions with high HMPO consumption, such as Guangzhou, routine liver function monitoring is essential. Guangzhou, situated in the south-central part of Guangdong Province, experiences a subtropical monsoon climate, with an average summer temperature of 28°C and an annual precipitation of 64 inches (26). Such warm and humid conditions favor the growth and toxin production of *Aspergillus flavus* in peanut raw materials. A previous study indicated severe AFB_1_ contamination in HMPO in Guangzhou (27). A subsequent food safety risk assessment identified HMPO as the primary dietary source of AFB_1_ exposure for residents in peripheral areas (14).

Small food workshops face challenges, including inadequate management and production inconsistencies, largely due to their limited scale, inconspicuous locations, and rudimentary production process. To enhance food safety management and protect public health, the Guangdong Provincial Government introduced the Small Food Workshops Management Regulations (SFWMR) (28). The regulations build on the Food Safety Law by providing clear guidelines for registering and supervising small food workshops. While existing study has primarily focused on the qualitative interpretation of the regulations (29–31), there has been no quantitative analyses of their implementation effects, particularly with respect to food contamination and consumer health. Our study addresses this gap by offering scientific evidence to evaluate the effectiveness of SFWMR in enhancing food safety and safeguarding public health.

In these contexts, our study aims to evaluate the impact of SFWMR implementation on AFB_1_ concentrations in HMPO and liver function among residents of the high-consumption area, and to explore the mediation effect of AFB_1_ concentrations in HMPO in the relation between SFWMR implementation and liver function.

## 2 Materials and methods

### 2.1 Sampling of home-made peanut oil

**Figure 1:**
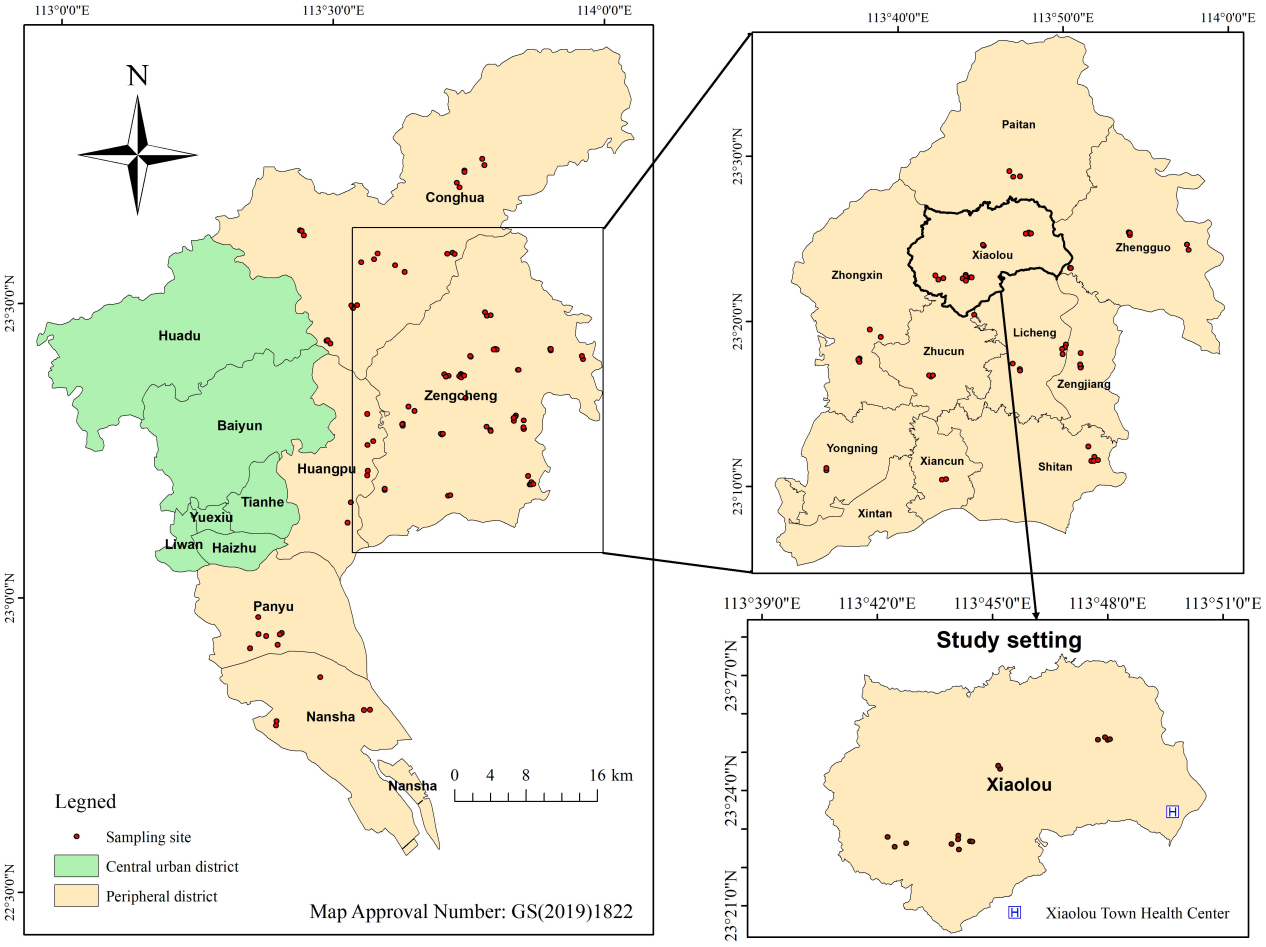
Distribution of sampling sites for home-made peanut oil from 2010 to 2022 and study setting.

Information on HMPO samples for this study was obtained from the Food Safety Risk Monitoring System of the Guangzhou Center for Disease Control and Prevention. HMPO consumption in Guangzhou is concentrated in peripheral districts. Sampling focused on the months of June to August, a period characterized by high temperatures and humidity. From 2010 to 2022, streets in peripheral districts were designated as sampling units, and street types were stratified based on their distance from the center of each district. Two central streets and one remote street were randomly selected as sampling sites in each peripheral area, resulting in 15 streets selected as sampling sites for HMPO. The distribution of sampling sites during the study period is presented in Figure 1. The sampling process involved two individuals, acting as consumers, procuring HMPO from small workshops to ensure the representativeness of the samples. A total of 590 HMPO samples were included in this study. After collection, the samples were sealed, refrigerated at 4°C, and analyzed within 48 h.

### 2.2 Detection of AFB_1_ in HMPO

The detection of AFB_1_ was conducted using high-performance liquid chromatography (HPLC) in accordance with the methodologies specified in Determination of Aflatoxins B_1_, B_2_, G_1_, and G_2_ in Foods (GB/T 5009.23-2006) (32) and Determination of Aflatoxins B and G Groups in Foods (GB 5009.22-2016) (33). The methodology outlined in GB 5009.22-2016 has been described in our previous study (14). The experimental procedures following GB/T 5009.23-2006 are as follows.

#### 2.2.1 Chemicals and instruments

Aflatoxin B_1_ standard (Purity > 98.0%, HPLC grade) was purchased from Sigma-Aldrich, USA; Acetonitrile, trifluoroacetic acid and hexane, all HPLC grade, were purchased from Merck, Germany; Ultrapure water; C18 reversed-phase column (Jiangsu Hming Technology Co., Ltd., China); A liquid chromatography system (SHIMADZU, Japan) equipped with RF-20A fluorescence detector; Milli-Q ultrapure water machine (Millipore, USA); Vortex mixer (IKA, Germany); Centrifuge (SIGMA, Germany); Nitrogen blower (Beijing Tongtailian Technology Development Co., Ltd., China); Electronic balance (METTLER-TOLEDO, USA).

#### 2.2.2 Sample extraction and purification

A 20.0 g aliquot of the HMPO sample was mixed with 80.0 mL of an acetonitrile-water (84:16) solution for 30 min, and then filtered through qualitative filter paper. An 8.0 mL portion of the filtrate was transferred to a multifunctional purification column for further processing. Subsequently, 2.0 mL of the purified solution was evaporated to dryness under nitrogen in a 60°C water bath. To the residue, 200.0 μL of hexane and 100.0 μL of trifluoroacetic acid were added, vortexed for 30 s, and derivatized at 40°C for 15 min. After derivatization, the sample was dried at room temperature, dissolved in 200.0 μL of a water-acetonitrile (85:15) solution, mixed and centrifuged. The supernatant was then collected for further analysis.

#### 2.2.3 Liquid chromatography condition

The mobile phase consisted of water and acetonitrile. Gradient elution was programmed as follows: starting with 15% acetonitrile at 0 min, increasing to 17% at 6 min, further increasing to 25% at 8 min, and returning to 15% at 14 min. A C18 reversed-phase column (125 mm × 2.1 mm, 5.0 μm particle size) was employed, with a flow rate of 0.5 mL/min and a column temperature of 30°C. The injection volume was 25.0 μL. Detection was performed at an excitation wavelength of 360 nm and an emission wavelength of 440 nm.

#### 2.2.4 Method validation

Weighed AFB_1_ standard and diluted with acetonitrile in a 10 mL volumetric flask to prepare a series of AFB_1_ standard working solutions at concentrations of 0.50, 2.50, 5.00, 25.00, 50.00, and 100.00 μg/L. The standard curve was expressed as y = 0.213x + 0.085, with an *R*-squared value of 0.9992 (Supplementary Figure S1).

The sensitivity of the method was assessed by determining the limit of detection (LOD) and limit of quantification (LOQ). The LOD and LOQ were 0.10 μg/kg and 0.30 μg/kg, determined at 3 times and 10 times the signal-to-noise ratio (S/N), respectively.

The spiked recovery experiment was conducted using blank samples. Six portions of each blank sample were spiked with low (5.00 μg/kg), intermediate (25.00 μg/kg) and high (50.00 μg/kg) levels of the AFB_1_ standard. The average spiked recovery percentages ranged from 90.48 to 97.27%, with relative standard deviations (RSDs) of 1.92–3.24%, meeting the requirements for trace analysis (Supplementary Table S1).

#### 2.2.5 Data processing

AFB_1_ detection values exceeding the acceptable limit of 20.0 μg/kg were regarded as exceeding the standard (34), and values below LOD were categorized as non-detect (ND). If the proportion of ND values was < 60%, these values were replaced with half of the LOD. Conversely, if the proportion of ND values was ≥60%, the values were substituted with the LOD (35).

### 2.3 Dietary exposure analysis

The deterministic exposure assessment model was employed to calculate the estimated dietary intake (EDI) of AFB_1_ through HMPO. *C* represents the AFB_1_ concentrations detected in HMPO samples; *IR* represents the daily intake of edible oil in the population, estimated from the consumption data in the Guangzhou Statistical Yearbook (36); *BW* represents the average body weight. According to the Report on Nutrition and Chronic Diseases of the Chinese Population, the average body weight was 66.2 kg for males and 57.3 kg for females (37). After applying the sex ratio of the Guangzhou population (38), the average body weight was determined to be 62.0 kg. The equation is as follows:


(1) $$\begin{array}{l} \begin{array}{c} EDI\left(ng/kg\;bw/d\right)\;=\;\frac{IR\;\left(g/d\right)\times C\;\left(\mu g/kg\right)}{BW\left(kg\right)} \end{array}\; \end{array}$$

### 2.4 Margin of exposure

Risk characterization for genotoxic and carcinogenic compounds, such as AFB_1_, is based on the margin of exposure (MOE), calculated by dividing 10% of the lower confidence limit of the benchmark dose (BMDL_10_) of AFB_1_ by the estimated dietary intake, as shown in Equation 2. The BMDL_10_ for AFB_1_ is 400 ng/kg bw/d (39). A smaller MOE indicates a greater risk of exposure to genotoxic and carcinogenic compounds. The European Food Safety Authority considers BMDL_10_ to be derived from animal studies. Due to inherent uncertainties, the MOE ≥ 10,000 indicates a low public health concern and a low priority for risk management (40).


(2) $$\begin{array}{l} \begin{array}{c} MOE\;=\;\frac{{BMDL}_{10}}{EDI} \end{array} \end{array}$$

### 2.5 Setting and collection of population data

The study setting was Xiaolou Town, Zengcheng District, Guangzhou. Xiaolou is a traditional agricultural town with a population of ~30,000 residents (41). Figure 1 and Supplementary Table S2 demonstrate the presence of numerous HMPO workshops in the area, establishing it as a representative high-HMPO-consumption region in Guangzhou. The Xiaolou Town Health Center is the sole health institution designated to provide the National Basic Public Health Service Program in the town (42) and offers annual health check-ups to residents. The study included participants from 20 local administrative villages, ensuring broad representativeness. Health data for residents from 2010 to 2022 were retrieved from the medical examination system of Xiaolou Town Health Center, and study variables included gender, age and liver enzyme levels. Individuals with a history of hepatitis B were excluded from the study.

AFB_1_ exposure is strongly associated with abnormal liver function, as indicated by elevated blood levels of liver enzymes, particularly aspartate aminotransferase (AST) and alanine aminotransferase (ALT) (43–45). These enzymes are abundant in liver cells and are released into the bloodstream upon liver cell damage, serving as bioindicators of abnormal liver function (46, 47). Liver function abnormality was defined according to the Reference Intervals for Common Clinical Biochemistry Tests (WS/T 404) as elevated AST or ALT levels, with AST > 40 U/L or ALT > 50 U/L for males and AST > 35 U/L or ALT > 40 U/L for females (48).

### 2.6 Statistical analysis

Interrupted Time Series Analysis (ITSA) is a statistical method used to evaluate the effectiveness of interventions through segmented regression models. Small Food Workshops Management Regulations have been in effect since October 1, 2015 (28). Considering the lag in the implementation effects and the annual nature of the data analyzed, the year 2016 was designated as the intervention breakpoint.

The continuous outcome variable was analyzed using a segmented linear regression model. The regression equation is as follows:


(3) $$\begin{array}{l} \begin{array}{c} Y_{t}\;=\;\beta_{0}+\beta_{1}T_{t}+\beta_{2}X_{t}+\beta_{3}\left(T_{t}-T\right)X_{t}+\varepsilon_{t} \end{array}\; \end{array}$$

In Equation 3, *Y*_*t*_ represents the outcome variable, which in this study denotes the AFB_1_ contamination in HMPO. *T*_*t*_ is a count variable for the year. *X*_*t*_ is an indicator variable for the intervention, with a value of 0 before the implementation of SFWMR and 1 after. (*T*_*t*_−*T*) represents the difference between the year count variable and the intervention breakpoint. ε_*t*_ is the random error term. The values assigned to these variables are detailed in Supplementary Table S3. β_0_ is the intercept. β_1_ indicates the trend before the intervention. β_2_ represents the level change after the intervention. β_3_ represents the difference in the slope between pre- and post-intervention periods. (β_1_+β_3_) reflects the trend after the intervention. We fitted both an unadjusted model and a model adjusted for potential confounders, including average annual temperature, rainfall, sunlight, and peanut yield.

Considering that the prevalence of liver function abnormality in this study was >10%, the binary outcome variable was analyzed using the log-binomial regression model (49). The regression equation is as follows:


(4) $$\begin{array}{l} \begin{array}{c} ln\left[P\left(Y_{t}\;=\;1\right)\right]\;=\;\beta_{0}+\beta_{1}T_{t}+\beta_{2}X_{t}+\beta_{3}\left(T_{t}-T\right)X_{t}+\varepsilon_{t} \end{array}\; \end{array}$$

(5) $$\begin{array}{l} \begin{array}{c} {PR}_{t}\;=\;\frac{\exp\left({\hat{\beta }}_{0}+{\hat{\beta }}_{1}T_{t}+{\hat{\beta }}_{2}+{\hat{\beta }}_{3}\left(T_{t}-T\right)\right)}{\exp\left({\hat{\beta }}_{0}+{\hat{\beta }}_{1}T_{t}\right)}\;=\;\exp\left({\hat{\beta }}_{2}+{\hat{\beta }}_{3}\left(T_{t}-T\right)\right) \end{array}\; \end{array}$$

(6) $$\begin{array}{l} \begin{array}{c} PR\;=\;{\left(\prod_{T}^{T+n}{PR}_{t}\right)}^{\frac{1}{n+1}}\;=\;\exp\left({\hat{\beta }}_{2}+\frac{n}{2}\;{\hat{\beta }}_{3}\right) \end{array} \end{array}$$

In Equation 4, *Y*_*t*_ represents whether the resident has liver function abnormality. The prevalence ratio (*PR*) was employed to quantify the intervention effect in the segmented log-binomial regression model. The *PR* was calculated by comparing the post-intervention fitted values with the counterfactual outcomes. Specifically, the prevalence ratio *PR*_*t*_, at each time point during the intervention period was calculated, and the geometric mean of *PR*_*t*_ was used to obtain the overall *PR* for the intervention period (50). Furthermore, stratified analyses were conducted by gender and age ≥ 60 years, with age stratification following the definition of older adults as stated by the World Health Organization (51). The Durbin-Watson method was used to detect first-order autocorrelation in the ITSA model. In the presence of autocorrelation, the Newey-West method was applied to adjust the standard errors of the parameter estimates.

Mediation analysis was conducted to investigate the role of AFB_1_ contamination in HMPO as a mediator of the relationship between the implementation of SFWMR and liver function abnormality in residents. The formula is as follows:


(7) $$\begin{array}{l} \begin{array}{c} \ln\left[P\left(Y\;=\;1|X,M,C\right)\right]\;=\;\theta_{0}+\theta_{1}X+\theta_{2}M+\theta_{3}C \end{array}\; \end{array}$$

(8) $$\begin{array}{l} \begin{array}{c} E\left[M|X,C\right]\;=\;\beta_{0}+\beta_{1}X+\beta_{2}C \end{array}\; \end{array}$$

(9) $$\begin{array}{l} \begin{array}{c} {PR}_{DE}\;=\;e^{\theta_{1}}{\;PR}_{IE}\;=\;e^{\left(\beta_{1}\theta_{2}\right)} \end{array}\; \end{array}$$

Where *Y* represents whether the resident has liver function abnormality. *X* indicates whether the resident was exposed to the implementation of SFWMR. *M* represents the annual AFB_1_ contamination in HMPO. *C* refers to potential confounding factors, including gender and age. The direct effect (DE) and indirect effect (IE) were described using the prevalence ratio, with the calculation of *PR* detailed in Equation 9. Mediation effects were evaluated using 1,000 bootstrap samples.

Microsoft Excel 2021 was employed for data organization and cleaning. Categorical variables were summarized as frequency and percentage. Continuous variables with a normal distribution were summarized using mean and standard deviation, while those with a skewed distribution were reported as median and interquartile range. All statistical analyses were conducted in R (version 4.1.3). *P* < 0.05 (two-tailed) was considered statistically significant.

## 3 Results

### 3.1 Contamination of AFB_1_ in HMPO

**Figure 2:**
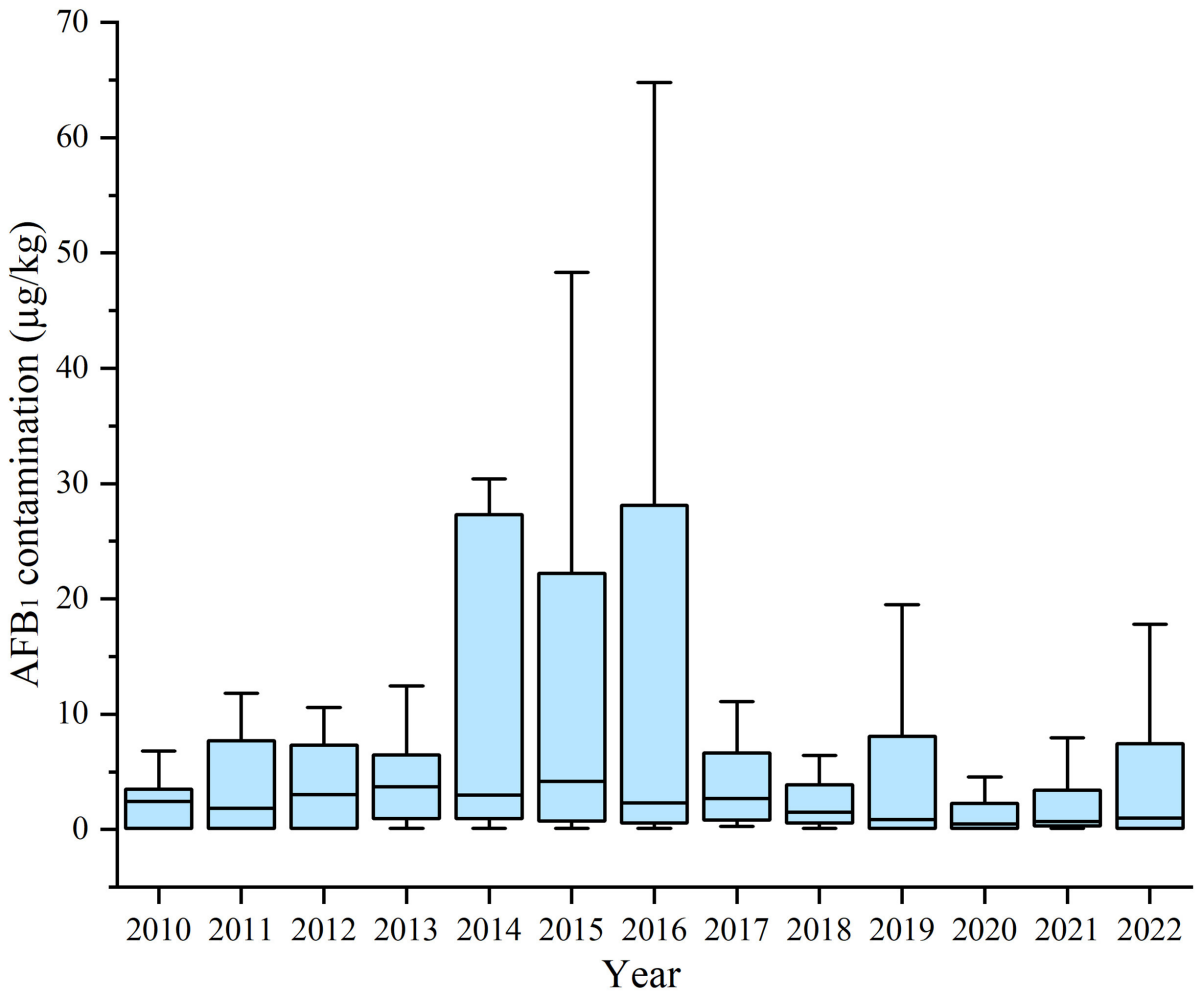
AFB_1_ contamination in home-made peanut oil in Guangzhou from 2010 to 2022.

The AFB_1_ contamination in HMPO were 1.29 (0.12, 6.58) μg/kg. Additionally, 67 samples exceeded the AFB_1_ standard, resulting in an exceedance rate of 11.36%. The variation in AFB_1_ contamination in HMPO between years was statistically significant (*P* = 0.019). After 2016, the AFB_1_ contamination in HMPO decreased from 2.72 (0.49, 7.47) μg/kg to 0.98 (0.12, 6.30) μg/kg (*P* = 0.011) (Figure 2; Supplementary Table S4).

### 3.2 EDI and MOE

**Figure 3:**
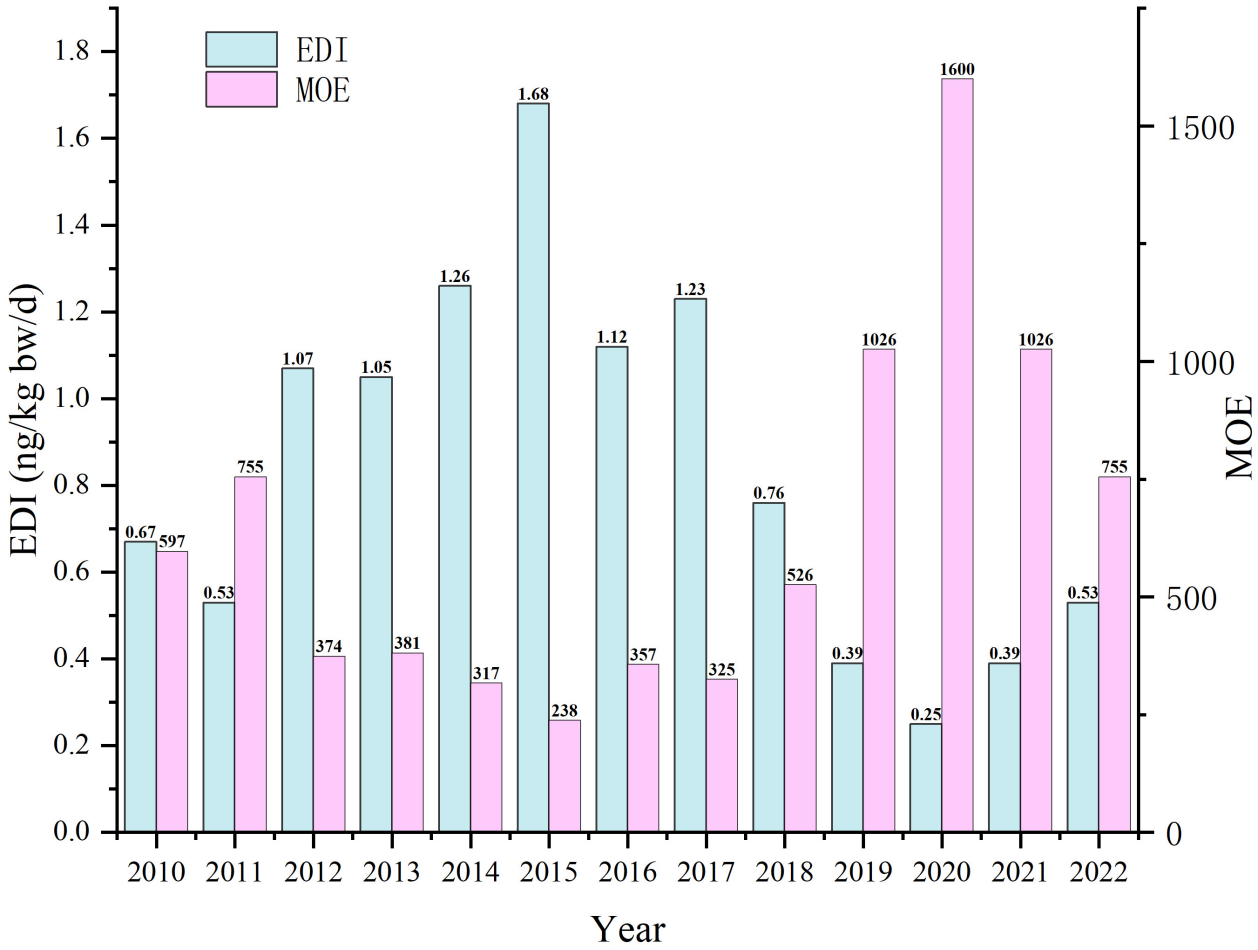
Estimated dietary intake of AFB_1_ through HMPO and margin of exposure in Guangzhou from 2010 to 2022.

As shown in Figure 3, the average daily intake of AFB_1_ through HMPO among Guangzhou residents from 2010 to 2022 ranged from 0.25 to 1.68 ng/kg bw/d. The MOE ranged from 238 to 1,600, which was lower than 10,000.

### 3.3 Liver function information of population

**Figure 4:**
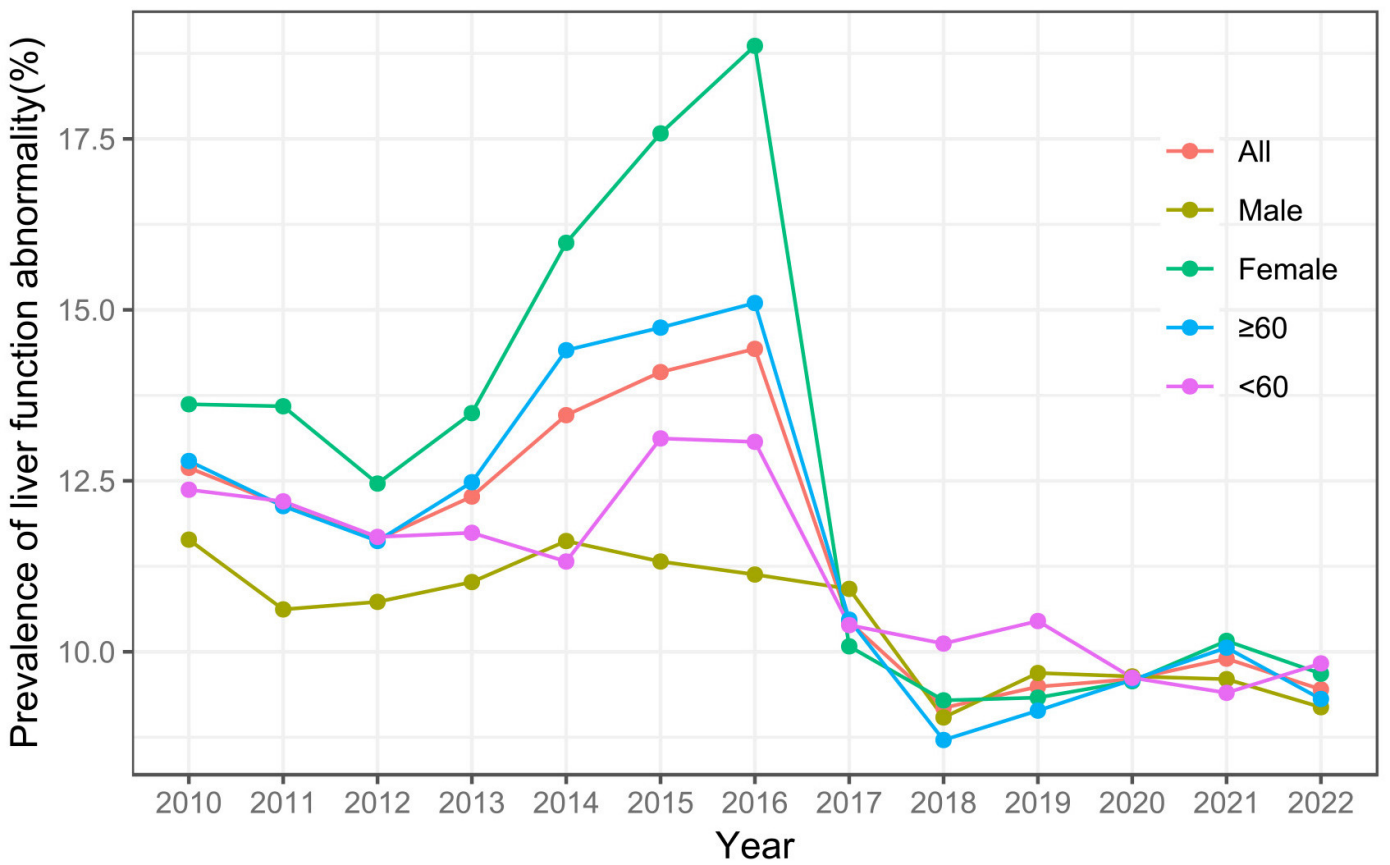
Temporal distribution of prevalence of liver function abnormality from 2010 to 2022.

The study included 21,828 residents, comprising 11,558 females (52.95%) and 10,270 males (47.05%). A total of 15,501 (71.01%) participants were aged ≥ 60 years and 6,327 (28.99%) were aged < 60 years. The mean age of the population was 64.34 ± 13.43 years. Of these residents, 2,289 (10.49%) were identified with liver function abnormality (Supplementary Table S5). Prior to 2016, both females, and individuals aged ≥ 60 years exhibited a higher prevalence of liver function abnormality, with an increasing trend. After 2016, the prevalence of liver function abnormality decreased and stabilized across all groups (Figure 4).

### 3.4 ITSA of AFB_1_ Levels in HMPO

**Figure 5:**
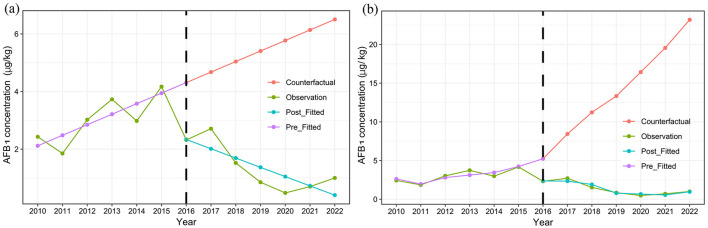
Trend of changes in AFB_1_ concentration in HMPO before and after the implementation of SFWMR. **(A)** is Model 1 and **(B)** is Model 2.

According to the adjusted model, prior to the implementation of SFWMR, AFB_1_ contamination in HMPO exhibited an increasing annual trend of 0.634 μg/kg (*P* = 0.013). In the first year following the implementation of SFWMR, AFB_1_ levels in HMPO decreased by 2.865 μg/kg (*P* = 0.006). After the implementation, there was a continued annual decrease in the AFB_1_ levels of 2.593 μg/kg (*P* = 0.034). Compared to the counterfactual scenario, the trend decreased by 3.227 μg/kg per year (*P* = 0.024) (Table 1; Figure 5).

**Table 1:** Result of the ITSA for AFB_1_ contamination in HMPO.

**Variables**	**Model 1**		**Model 2**	
	**Coefficient (95%CI)**	***P***	**Coefficient (95%CI)**	***P***
Baseline trend (*β1*)	0.366 (0.071, 0.660)	**0.020**	0.634 (0.200, 1.067)	**0.013**
Level change (*β2*)	−1.975 (−3.396, −0.554)	**0.012**	−2.865 (−4.450, −1.279)	**0.006**
Trend change (*β3*)	−0.688 (−1.063, −0.313)	**0.003**	−3.227 (−5.820, −0.634)	**0.024**
Trend after intervention (*β1+β3*)	−0.322 (−0.555, −0.089)	**0.012**	−2.593 (−4.893, −0.294)	**0.034**
Temperature	–	–	−0.551 (−1.824, 0.722)	0.316
Precipitation	–	–	4.240 × 10^−4^ (−1.218 × 10^−3^, 2.065 × 10^−3^)	0.536
Sunshine	–	–	−3.780 × 10^−4^ (−3.884 × 10^−3^, 3.128 × 10^−3^)	0.793
Peanut yield	–	–	−1.579 × 10^−3^ (−3.114 × 10^−3^, −4.286 × 10^−5^)	**0.046**

Model 2 adjusted the annual average temperature, precipitation, sunshine, and peanut yield based on Model 1. Model 1 (DW = 2.140, *P* = 0.216; *R*^2^ = 0.8415, Adjusted *R*^2^ = 0.7887); Model 2 (DW = 2.617, *P* = 0.509; *R*^2^ = 0.9384, Adjusted *R*^2^ = 0.8522). Bold values mean *p*-value < 0.05.

### 3.5 ITSA of liver function in population

**Figure 6:**
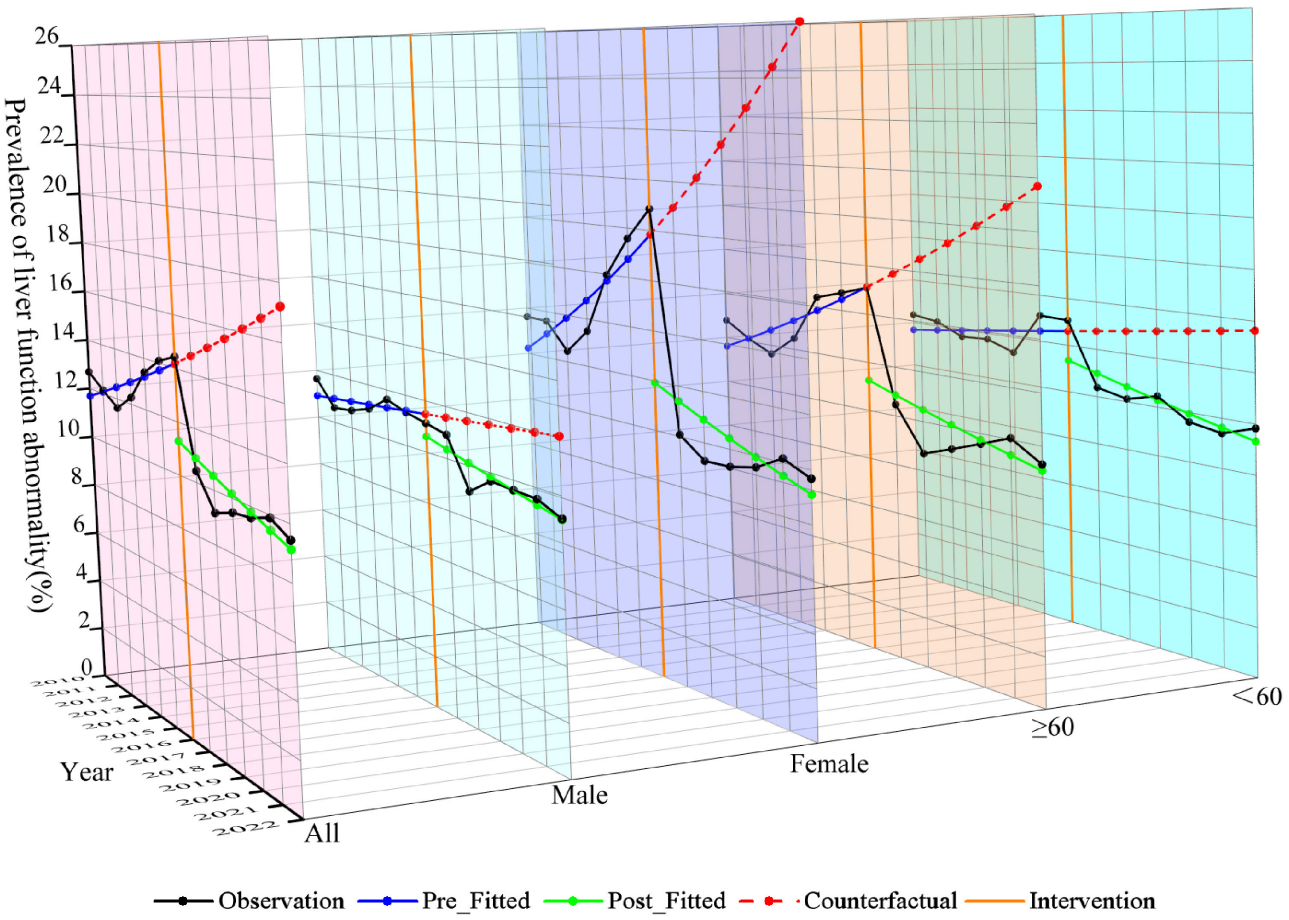
Trend of changes in prevalence of liver function abnormality among population before and after the implementation of SFWMR.

**Figure 7:**
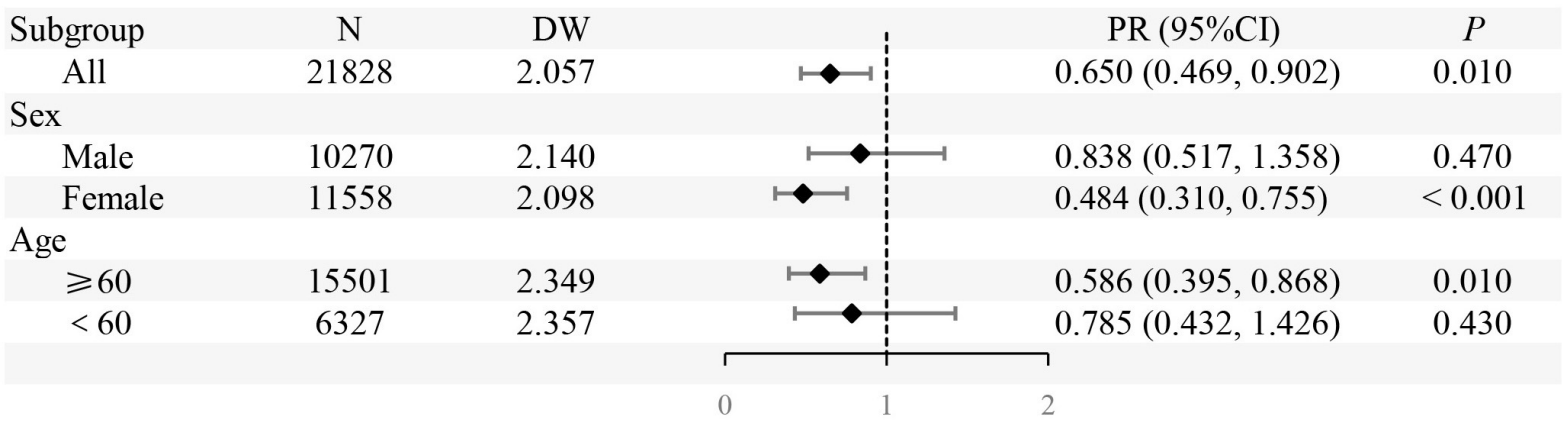
The prevalence ratio (PR) of prevalence of liver function abnormality in different subgroups before and after the implementation of SFWMR compared to the counterfactual outcome.

In the entire population, the implementation of SFWMR was associated with a reduction in the prevalence of liver function abnormality compared to the counterfactual outcome (PR = 0.650, 95% CI: 0.469–0.902). When stratified by gender and age, significant associations were observed in both the female group (PR = 0.484, 95% CI: 0.310–0.755) and the age ≥ 60 years group (PR = 0.586, 95% CI: 0.395–0.868). However, no significant associations were found in the male group or among participants aged < 60 years (Figures 6, 7).

### 3.6 Mediation analysis

As shown in Table 2, the implementation of SFWMR had a total effect on liver function abnormality (PR = 0.970, 95% CI: 0.959–0.981). The analysis revealed an indirect effect of AFB_1_ contamination in HMPO on the relationship between the implementation of SFWMR and the liver function abnormality (PR = 0.981, 95% CI: 0.969–0.993). AFB_1_ contamination in HMPO fully mediated the relationship between the implementation of SFWMR and liver function abnormality.

**Table 2:** Result of mediation analysis.

	**PR**	**95% CI**	***P***
Indirect effect	0.981	(0.969, 0.993)	0.004
Direct effect	0.989	(0.972, 1.005)	0.214
Total effect	0.970	(0.959, 0.981)	< 0.001

## 4 Discussion

### 4.1 AFB_1_ contamination of HMPO

Aflatoxin contaminants in food are widely recognized as a significant health concern (52). In this study, AFB_1_ was detected in HMPO samples from Guangzhou from 2010 to 2022 by HPLC, with an exceedance rate of 11.36%. This rate is comparable to the national average of 11.11% (53) and lower than rates reported in other areas of Guangdong Province, including Huizhou (19.72%) (54), Heyuan (11.82%) (55), and the western region of Guangdong (18.7%) (15). This variability in regulatory enforcement could partly explain the disparities in exceedance rates between Guangzhou and other cities in Guangdong Province. While the SFWMR specifies the requirements for obtaining a food workshop registration certificate, the specific application processes, inspection criteria, and acceptance scopes are tailored by each prefecture-level city according to their local circumstances (56).

However, the exceedance rate in Guangzhou is higher than the rate reported in Tianjin (6.67%) (57). This discrepancy may be attributed to regional differences in climate and soil characteristics. Guangzhou's warm and humid subtropical climate provides optimal conditions for the growth of *Aspergillus* molds that produce toxins (58). Moreover, the predominantly clay-based soil in Guangzhou contrasts with the sandy soil prevalent in Tianjin (59). The high water retention capacity of clay facilitates the growth of *Aspergillus* species (60), thereby increasing the risk of peanut contamination before harvesting. Contaminated peanuts introduce AFB_1_ into the product during the pressing process (61).

### 4.2 AFB_1_ dietary exposure and MOE

Dietary intake is the main route of AFB_1_ exposure. Our study observed that the average daily intake of AFB_1_ through HMPO ranged from 0.25 to 1.68 ng/kg bw/d for Guangzhou residents. The MOEs were all below 10,000, consistent with the findings of HE, indicating that the health risks associated with HMPO warrant attention (25). A deterministic exposure assessment was performed based on the general population in our study. Notably, age was found to be an important factor influencing the EDI of AFB_1_, with children experiencing the highest exposure risk (14, 62). Given their higher exposure levels and the potential for synergistic hepatotoxic effects from chronic AFB_1_ intake, coupled with their relatively immature immune systems, children are particularly vulnerable (63).

### 4.3 Impact of SFWMR on AFB_1_ contamination in HMPO

Based on the ITSA, we evaluated the time-variant impact of SFWMR on AFB_1_ contamination in HMPO. In the counterfactual scenario, AFB_1_ contamination in HMPO would have increased yearly. Aflatoxin contamination in food is predominantly due to improper handling of raw materials (64). A study in Kenya showed that the source of peanuts and the presence of defective nuts were the primary contributors to increased aflatoxin contamination in the cottage industry (65). Small workshops often purchase large quantities of peanut raw materials at one time. Their storage facilities lack temperature control and dehumidification systems, rendering peanuts highly susceptible to mold contamination. Additionally, these workshops often fail to effectively remove moldy peanuts and do not perform oil refining treatments (55). A research demonstrated that the refining process can significantly reduce AFB_1_ content in peanut oil (66). Thus, incorporating the refining process into the standard production of HMPO is recommended.

After the implementation of SFWMR, there was a significant reduction in AFB_1_ contamination in HMPO, and this downward trend persisted in subsequent years. This decrease can be attributed to the impact of SFWMR. For instance, article 14 of the regulations explicitly prohibits small food workshops from producing edible oils that do not meet food safety standards and defines penalties for non-compliance. Secondly, the regulations standardize the registration process of small food workshops by requiring on-site inspection of storage facilities, production environments, layout of functional areas, refining equipment, and product labels. Workshops must also provide qualified AFB_1_ inspection reports for HMPO issued by the third-party testing institution during the inspection. Furthermore, the regulations establish the production license publicity system, which promotes the healthy development of small food workshops by leveraging market forces through consumer choice (28). Our study emphasized the positive impact of these comprehensive intervention measures in standardizing HMPO production in small food workshops. In Africa, small-scale operators and unorganized markets present key challenges to effective mycotoxin regulation (67). The practices implemented in Guangzhou may serve as a reference for these regions.

### 4.4 Impact of SFWMR on liver function in the population

Aflatoxin contamination in HMPO in Guangdong urgently requires urgent attention, highlighting the need for enhanced public health management of consumers (25). The study evaluated the potential health impact of SFWMR in a region characterized by high HMPO consumption, focusing on liver function. Our findings indicated that the implementation of SFWMR was associated with a decrease in the prevalence of liver function abnormality. Moreover, mediation analysis revealed that the protective effect of SFWMR implementation on liver function operates through the reduction of AFB_1_ contamination in HMPO. From the disease prevention perspective, the implementation of SFWMR reduced AFB_1_ exposure and played the primary preventive role in improving liver function in the population. Evidence from animal experiments and epidemiologic studies demonstrates an association between exposure to AFB_1_ and abnormal liver function. In rats, chronic exposure to AFB_1_ resulted in hepatocellular damage and significantly elevated serum levels of liver enzymes and oxidative stress markers (46). Population-based studies in Saudi Arabia and Ghana similarly reported a significant association between AFB_1_ exposure and liver function enzyme levels (45, 68). In addition, AFB_1_ exposure acts synergistically with hepatitis B virus infection to cause liver dysfunction (25). Mechanistically, the hepatotoxicity of AFB_1_ arises from its active metabolite, AFB_1_-8,9-epoxide, which induces mitochondrial dysfunction, oxidative stress, and inflammatory responses, ultimately leading to apoptosis and necrosis of hepatocytes (17).

Additionally, subgroup analysis revealed that the implementation of SFWMR resulted in significant improvements in liver function for females and individuals aged ≥ 60 years. This improvement may be attributed to consumer habits. HMPO, which has a lower oil yield, is infrequently used by commercial food service establishments, but is preferred by females and older adults for home cooking. Males and younger individuals, particularly those working outdoors, tend to consume less HMPO. This preference aligns with Mills' findings, which indicate that females and older individuals are more likely to consume homemade meals (69). Consequently, after the implementation of SFWMR, AFB_1_ intake among females and older adults significantly decreased, resulting in a more pronounced protective effect on liver function. AFB_1_ exposure is not only related to food contamination but also to consumption. This highlights the need for greater attention to high-HMPO-consumption groups.

### 4.5 Significance of SFWMR

Small food workshops face supervision challenges due to their low entry thresholds, broad distribution, and mobility. Despite the enactment of the National Food Safety Standard for Maximum Levels of Mycotoxins in Food in China, supervising small food workshops remains problematic (15). These small workshops are crucial for supporting local agriculture and sideline products, addressing employment issues, and increasing residents' incomes. They also contribute significantly to preserving regional characteristics and dietary culture. The implementation of SFWMR is not a restriction on entrepreneurial freedom. On the contrary, it provides a framework for standardizing productions and encourages small food workshops to upgrade and renovate their facilities to obtain food production licenses. The implementation of SFWMR address legislative and regulatory gaps for small food workshops, enhancing food safety through legal thinking and methods (70).

### 4.6 Limitation

The study analyzed the implementation effects of SFWMR from the perspective of food contamination and the health impacts on consumers. However, our study had several limitations. Firstly, although ALT and AST are crucial for liver function assessment and hepatocellular damage monitoring, their elevation may also indicate non-hepatic tissue damage, potentially leading to false positives (71). To enhance the specificity of liver disease diagnosis, other biomarkers and imaging tests can be used in tandem. Gamma-glutamyltransferase (GGT) and Alkaline phosphatase (ALP) exhibit distinct expression patterns compared to ALT and AST in liver disease (44). GGT, concentrated in the liver and biliary system, often rises in cholestatic diseases and liver injury. ALP, present in the hepatobiliary system, bones, placenta, and intestines, may indicate bone or liver disease (47). Additionally, imaging tests such as ultrasound, Computed Tomography scans, and Magnetic Resonance Imaging are important tools for assessing liver function. They provide visual information about the structure and morphology of the liver and help identify the presence and progression of liver diseases. The combined use of these biomarkers and imaging tests can help differentiate liver from non-liver diseases and improve diagnostic accuracy (72). Secondly, the liver function data were obtained from the health examination system, which may introduce selection bias. Finally, although the single-group ITSA method used in this study provides reliable inferences, the ecological study design limits the determination of causality. Future follow-up studies will address these limitations by incorporating additional biochemical and imaging tests, increasing the sample size and establishing a control group to more comprehensively evaluate the effectiveness of SFWMR implementation.

## 5 Conclusion

In Guangzhou, the public health concern arising from AFB_1_ intake through HMPO requires considerable attention. The enactment of SFWMR significantly contributed to reducing AFB_1_ contamination in HMPO and improving liver function. These outcomes highlight the efficacy of regulatory interventions in addressing food safety hazards and promoting public health. Continued efforts are necessary to strengthen the enforcement of the regulations. The exposure risks to AFB_1_ among high-HMPO-consumption groups also demand greater focus.

## Data Availability

The raw data supporting the conclusions of this article will be made available by the authors, without undue reservation.
