# Supplementary material for: The impact of small food workshops management regulations on aflatoxin B1 in home-made peanut oil and the liver function of high-consumption area residents: an interrupted time series study in Guangzhou, China
Source: Front Public Health. 2024 Dec 20;12:1484414. doi: 10.3389/fpubh.2024.1484414 (PMC11695283; doi:10.3389/fpubh.2024.1484414)
Supplement: Supplementary file 1 [file Data_Sheet_1.docx]

Supplementary Material

# Supplementary Figures and Tables

## Supplementary Figures


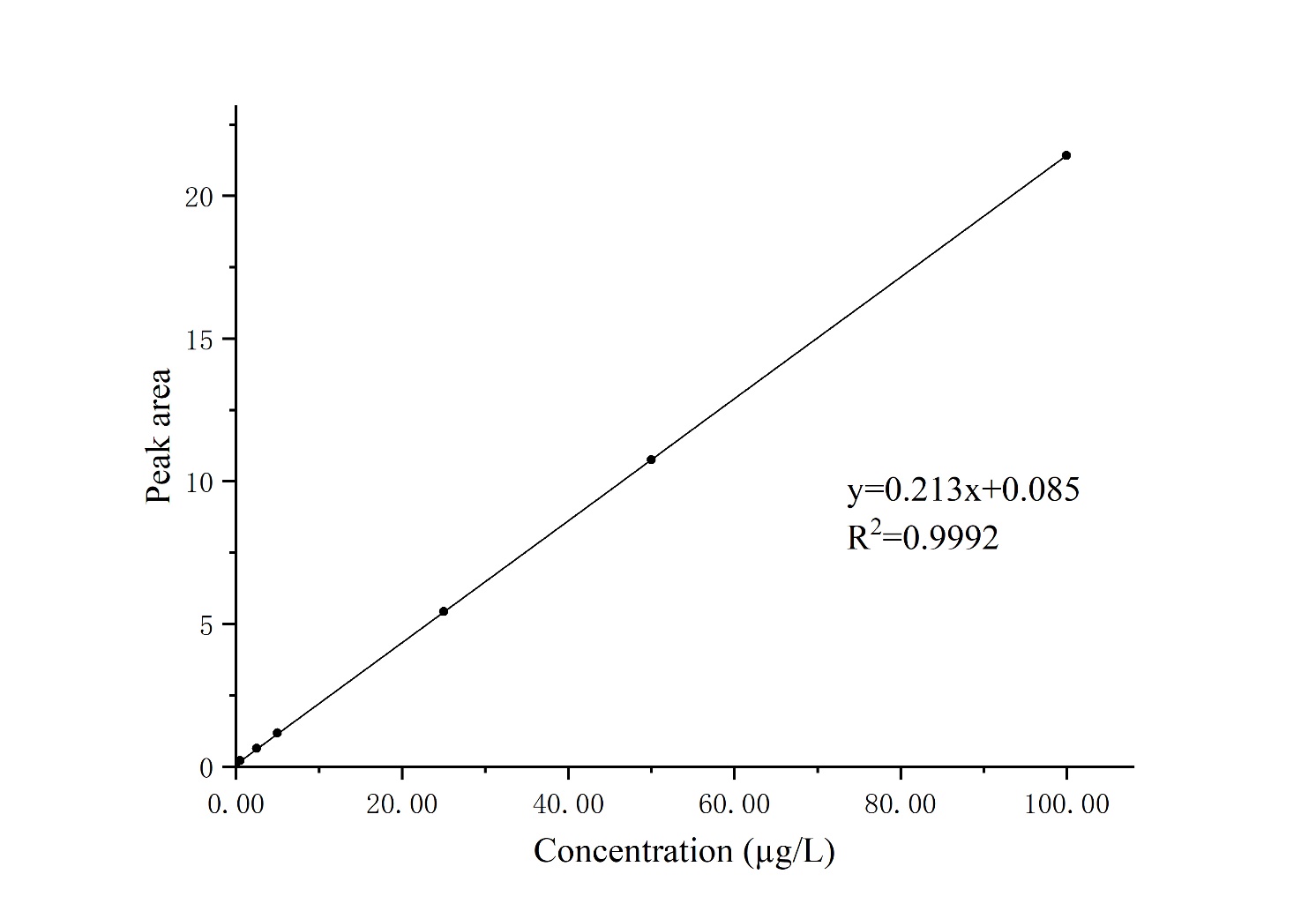


Figure S1. AFB_1_ Standard Curve

## Supplementary Tables

Table S1. Result of Spiking Recovery Experiment

| Spiked concentration levels (µg/kg) | Measured value (µg/kg) | Spiked recovery percentage (%) | Average spiked recovery percentage (%) | Standard deviation | RSD(%) |
| --- | --- | --- | --- | --- | --- |
| 5 | 4.78 | 95.60 | 97.27 | 2.25 | 2.31 |
|  | 4.92 | 98.40 |  |  |  |
|  | 4.90 | 98.00 |  |  |  |
|  | 4.73 | 94.60 |  |  |  |
|  | 4.81 | 96.20 |  |  |  |
|  | 5.04 | 100.8 |  |  |  |
| 25 | 23.91 | 95.62 | 90.48 | 2.93 | 3.24 |
|  | 22.86 | 91.45 |  |  |  |
|  | 21.88 | 87.52 |  |  |  |
|  | 22.70 | 90.80 |  |  |  |
|  | 22.04 | 88.14 |  |  |  |
|  | 22.34 | 89.34 |  |  |  |
| 50 | 46.05 | 92.10 | 92.44 | 1.78 | 1.92 |
|  | 44.88 | 89.76 |  |  |  |
|  | 46.72 | 93.44 |  |  |  |
|  | 45.58 | 91.15 |  |  |  |
|  | 47.28 | 94.56 |  |  |  |
|  | 46.80 | 93.60 |  |  |  |

Table S2. Number of Homemade Peanut Oil Workshops in Guangzhou by District

| District | Town or stree | Number of small HMPO workshops |
| --- | --- | --- |
| Conghua | Aotou town | 4 |
|  | Taiping town | 6 |
|  | Chengjiao street | 3 |
|  | Jiangpu street | 2 |
|  | Wenquan town | 3 |
|  | Liangkou town | 4 |
|  | Liuxihe forestry | 2 |
| Zengcheng | Paitan town | 3 |
|  | Zhongxin town | 7 |
|  | Xiaolou town | 15 |
|  | Zhengguo town | 7 |
|  | Zhucun street | 3 |
|  | Licheng street | 8 |
|  | Zengjiang street | 5 |
|  | Yongning street | 2 |
|  | Xiancun town | 2 |
|  | Shitan town | 5 |
| Huangpu | Jiulong town | 3 |
|  | Yunpu street | 2 |
|  | Yonghe street | 2 |
| Panyu | Shawan town | 1 |
|  | Qiaonan street | 1 |
|  | Shiqiao street | 2 |
|  | Dalong street | 2 |
|  | Donghuan street | 1 |
| Nansha | Dagang town | 2 |
|  | Dongyong town | 1 |
|  | Nansha street | 2 |
| Other District |  | 0 |

Table S3. Description of ITSA variables assignment

| Year | $T_{t}$ | $X_{t}$ | $T_{t}-T$ |
| --- | --- | --- | --- |
| 2010 | 1 | 0 | -6 |
| 2011 | 2 | 0 | -5 |
| 2012 | 3 | 0 | -4 |
| 2013 | 4 | 0 | -3 |
| 2014 | 5 | 0 | -2 |
| 2015 | 6 | 0 | -1 |
| 2016 | 7 | 1 | 0 |
| 2017 | 8 | 1 | 1 |
| 2018 | 9 | 1 | 2 |
| 2019 | 10 | 1 | 3 |
| 2020 | 11 | 1 | 4 |
| 2021 | 12 | 1 | 5 |
| 2022 | 13 | 1 | 6 |

Table S4. AFB_1_ contamination in home-made peanut oil from 2010 to 2022

| Year | N | Detected (%) | Exceed (%) | M (P25, P75) | *P* |
| --- | --- | --- | --- | --- | --- |
| 2010 | 15 | 11 (73.33) | 1 (6.67) | 2.43 (0.34, 3.27) | 0.019^a^ |
| 2011 | 15 | 10 (66.67) | 1 (6.67) | 1.85 (0.12, 5.62) |  |
| 2012 | 18 | 12 (66.67) | 2 (11.11) | 3.02 (0.12, 7.13) |  |
| 2013 | 26 | 22 (84.62) | 2 (7.69) | 3.73 (0.98, 6.24) |  |
| 2014 | 20 | 15 (75.00) | 6 (30.00) | 2.98 (1.37, 25.77) |  |
| 2015 | 20 | 16 (80.00) | 5 (25.00) | 4.17 (0.76, 18.66) |  |
| 2016 | 30 | 24 (80.00) | 8 (26.67) | 2.32 (0.61, 25.60) |  |
| 2017 | 20 | 20 (100.00) | 2 (10.00) | 2.71 (0.90, 6.40) |  |
| 2018 | 20 | 18 (90.00) | 3 (15.00) | 1.52 (0.57, 3.69) |  |
| 2019 | 60 | 40 (66.67) | 5 (8.33) | 0.85 (0.12, 7.72) |  |
| 2020 | 56 | 38 (67.86) | 3 (5.36) | 0.48 (0.12, 2.24) |  |
| 2021 | 80 | 64 (80.00) | 6 (7.50) | 0.70 (0.32, 3.19) |  |
| 2022 | 210 | 132 (62.86) | 23 (10.95) | 1.00 (0.12, 7.43) |  |
| <2016 | 114 | 86 (75.44) | 17 (14.91) | 2.72 (0.49, 7.47) | 0.011^b^ |
| ≥2016 | 476 | 336 (70.59) | 50 (10.50) | 0.98 (0.12, 6.30) |  |
| Total | 590 | 422 (71.53) | 67 (11.36) | 1.29 (0.12, 6.58) |  |

Note: N: Number of homemade peanut oil samples. M: Median, unit: µg/kg. ^a^ : Kruskal-Wallis rank sum test, Kruskal-Wallis chi-squared = 24.281；^b^ : Wilcoxon rank sum test, W = 31,250.

Table S5. Liver function information of population from 2010 to 2022

|  | All | | Male | | Female | | ≥ 60 | | < 60 | |
| --- | --- | --- | --- | --- | --- | --- | --- | --- | --- | --- |
| Year | N | n (%) | N | n (%) | N | n (%) | N | n(%) | N | n (%) |
| 2010 | 402 | 51 (12.69) | 189 | 22 (11.64) | 213 | 29 (13.62) | 305 | 39 (12.79) | 97 | 12 (12.37) |
| 2011 | 560 | 68 (12.14) | 273 | 29 (10.62) | 287 | 39 (13.59) | 437 | 53 (12.13) | 123 | 15 (12.20) |
| 2012 | 550 | 64 (11.64) | 261 | 28 (10.73) | 289 | 36 (12.46) | 413 | 48 (11.62) | 137 | 16 (11.68) |
| 2013 | 750 | 92 (12.27) | 372 | 41 (11.02) | 378 | 51 (13.49) | 537 | 67 (12.48) | 213 | 25 (11.74) |
| 2014 | 862 | 116 (13.46) | 499 | 58 (11.62) | 363 | 58 (15.98) | 597 | 86 (14.41) | 265 | 30 (11.32) |
| 2015 | 951 | 134 (14.09) | 530 | 60 (11.32) | 421 | 74 (17.58) | 570 | 84 (14.74) | 381 | 50 (13.12) |
| 2016 | 1067 | 154 (14.43) | 611 | 68 (11.13) | 456 | 86 (18.86) | 715 | 108 (15.10) | 352 | 46 (13.07) |
| 2017 | 1714 | 179 (10.44) | 742 | 81 (10.92) | 972 | 98 (10.08) | 1127 | 118 (10.47) | 587 | 61 (10.39) |
| 2018 | 2026 | 186 (9.18) | 874 | 79 (9.04) | 1152 | 107 (9.29) | 1344 | 117 (8.71) | 682 | 69 (10.12) |
| 2019 | 2729 | 259 (9.49) | 1207 | 117 (9.69) | 1522 | 142 (9.33) | 1992 | 182 (9.14) | 737 | 77 (10.45) |
| 2020 | 3114 | 299 (9.60) | 1411 | 136 (9.64) | 1703 | 163 (9.57) | 2189 | 210 (9.59) | 925 | 89 (9.62) |
| 2021 | 3526 | 349 (9.90) | 1636 | 157 (9.60) | 1890 | 192 (10.16) | 2654 | 267 (10.06) | 872 | 82 (9.40) |
| 2022 | 3577 | 338 (9.45) | 1665 | 153 (9.19) | 1912 | 18 5(9.68) | 2621 | 244 (9.31) | 956 | 94 (9.83) |
| Total | 21828 | 2289 (10.49) | 10270 | 1029 (10.02) | 11558 | 1260 (10.90) | 15501 | 1623 (10.47) | 6327 | 666(10.53) |

Note: N is the total number of people in each group in that year, and n is the number of people with liver function abnormality in each group in that year.
